# Synthesis of Ag doped calcium phosphate particles and their antibacterial effect as additives in dental glass ionomer cements

**DOI:** 10.1007/s10856-016-5785-3

**Published:** 2016-10-04

**Authors:** Song Chen, Satwik Gururaj, Wei Xia, Håkan Engqvist

**Affiliations:** Applied Materials Science, Department of Engineering Science, Uppsala University, Uppsala, Sweden

## Abstract

Developing dental restorations with enhanced antibacterial properties has been a constant quest for materials scientists. The aim of this study was to synthesize silver doped calcium phosphate particles and use them to improve antibacterial properties of conventional glass ionomer cement. The Ag doped monetite (Ag-DCPA) and hydroxyapatite (Ag-HA) were synthesized by precipitation method and characterized using X-ray diffraction, scanning electron microscope and X-ray fluorescence spectroscopy. The antibacterial properties of the cements aged for 1 day and 7 days were evaluated by direct contact measurement using staphylococcus epidermis Xen 43. Ion concentrations (F^−^ and Ag^+^) and pH were measured to correlate to the results of the antibacterial study. The compressive strength of the cements was evaluated with a crosshead speed of 1 mm/min. The glass ionomer cements containing silver doped hydroxyapatite or monetite showed improved antibacterial properties. Addition of silver doped hydroxyapatite or monetite did not change the pH and ion release of F^−^. Concentration of Ag^+^ was under the detection limit (0.001 mg/L) for all samples. Silver doped hydroxyapatite or monetite had no effect on the compressive strength of glass ionomer cement.

## Introduction

Secondary caries is one of the most common reasons responsible for the replacement of dental restorations [1]. Caries is a biofilm-sugar dependent disease and the accumulation of oral bacteria in the dental restorations plays a key role in the development of secondary caries [2, 3]. Developing dental restorations with enhanced antibacterial properties has therefore been a constant quest for materials scientists. Some efforts have been made to improve the antibacterial properties of dental restorations, including incorporation of soluble/releasing antibacterial agents, polymerizable antibacterial components or inorganic fillers such as Ag and ZnO [4]. Although some of these methods are demonstrated effective in inhibiting bacteria, they show limitations due to their distinct disadvantages. E.g., release of antibacterial agents usually exhibits a burst effect and results in porous structure and inferior mechanical properties [5, 6]. Polymerizable antibacterial components are only used in resin matrix and they only kill bacteria that come into contact with resin materials [4], while other antibacterial components such as ZnO and Ag appear to be cytotoxic [7, 8]. Therefore development of new antibacterial agents that can enhance the antibacterial properties of dental restorations without compromising their mechanical properties and biocompatibility still remains a big challenge.

Recently calcium phosphate biomaterials have attracted much interest due to their chemical similarities to bone mineral. Among these calcium phosphate biomaterials, hydroxyapatite (HA, Ca_10_(PO_4_)_6_(OH)_2_)) is the main component of human bone and teeth while monetite (DCPA, CaHPO_4_,) is the least soluble calcium phosphate phases under acidic condition (pH below 4.8) [9]. Both HA and DCPA show good biocompatibility and bioactivity, which make them have broad applications in orthopedic and dental applications [10–12]. Despite the advantages of HA and DCPA, they do not show any antibacterial effects. Some research has been conducted to improve the antibacterial properties of HA [13–15]. More recently silver doped hydroxyapatite (Ag-HA) has been proposed as an antibacterial agent. Previous studies show that Ag-HA is effective in killing bacteria either as nano particles or as implant coatings [16–18]. However, to the best of our knowledge, synthesis of Ag doped monetite (Ag-DCPA) particles has not been reported and their potential as antibacterial agents is still unknown. In addition, whether Ag-HA and Ag-DCPA possess antibacterial effects when served as additives in dental cements is still unknown. Served as additives in the dental cements, the physical and chemical environment around these particles would be distinctly different from that of coatings. Thus it is our interest to investigate the potential of Ag-DCPA and Ag-HA as antibacterial agents in the dental cements. Therefore two types of Ag doped calcium phosphate particles (Ag-HA and Ag-DCPA) were synthesized in this study. Here one of the most widely used dental cements—glass ionomer cement (GIC) was chosen as control to investigate the antibacterial properties of Ag-HA and Ag-DCPA. The effect of Ag-HA and Ag-DCPA on the mechanical properties of GIC was also studied. Factors such as pH change, Ag^+^ and F^−^ concentration might affect the antibacterial properties of GIC, therefore, to understand the mechanism behind their antibacterial performance, these factors were also measured for all the samples.

## Materials and methods

### Synthesis of Ag-DCPA and Ag-HA particles

Ag-DCPA: Solution 1 was prepared by dissolving Ca(NO_3_)_2_·4H_2_O (Sigma-Aldrich, MKBK6090) to 0.294 M and AgNO_3_ (Sigma-Aldrich, 209139) to 0.006 M in distilled water. Solution 2 was prepared by dissolving (NH_4_)_2_HPO_4_ (ACROS organics, A0323475) to 0.18 M in equal amount of distilled water. Solution 2 was dropped into solution 1. The final solution was stirred at 110 °C for 2 h. The final product was washed 3 times using distilled ethanol and water.

Ag-HA: Solution 1 was prepared by dissolving Ca(NO_3_)_2_·4H_2_O to 0.49 M and AgNO_3_ to 0.01 M in 100 ml distilled water, 10 ml NH_3_·H_2_O was added to adjust the pH. Solution 2 was prepared by dissolving (NH_4_)_2_HPO_4_ to 0.3 M in equal amount of distilled water. Solution 2 was dropped into solution 1 and the final solution was stirred for 2 h. The final product was washed 3 times using distilled ethanol and water.

### Characterizations of Ag-DCPA and Ag-HA

X-ray diffraction (XRD) measurements were conducted by using a D8 diffractometer (Bruker, USA) with Cu Kα1 radiation (*λ* = 1.5418 Å). The step size was 0.02° and the scan speed was 2 s per step. A scanning electron microscope (SEM, LEO 1550, Zeiss, Germany) was used to analyze the morphologies of synthesized particles. The samples were coated with a thin gold/palladium layer to avoid surface charging. The elemental composition of the samples was analyzed on an X-ray fluorescence spectrometer (XRF, Epsilon 1, PANalytical, the Netherlands). The calcium phosphate particles were pressed to form a thin disc before XRF measurement.

### Preparation of cement samples

GIC (Advanced Healthcare Ltd, 101321-4) specimens were prepared in accordance with the manufacturer’s instructions. For Ag-HA and Ag-DCPA modified GIC, corresponding glass powder was replaced by Ag-HA or Ag-DCPA and mixed by a Turbula mixer (Willy A.Bachofen AG, Switzerland). The cements were mixed by hand using a stainless steel spatula on a clean surface. Five formulations were studied: (1) GIC Control (2) GIC modified by 10 % Ag-DCPA (3) GIC modified by 20 % Ag-DCPA (4) GIC modified by 10 % Ag-HA (5) GIC modified by 20 % Ag-HA. Poly(methyl methacrylate) (PMMA, Surgical simplex P, stryker, USA), which is considered inert, was used as control when conducting direct contact test.

### Direct contact test

Staphylococcus epidermidis Xen 43, a bioluminescent strain, was used to evaluate the antibacterial activities of cements by direct contact test. In direct contact test all the bacteria are allowed to stay on the samples and proliferate. The antibacterial effect of the material can be seen more evidently by this method. Briefly, staphylococcus epidermidis Xen 43 was inoculated in tryptic soy broth (TSB) and cultured in 37 °C oven for 18 h. The bacteria pellet was obtained by centrifuging the culture at 2000 rpm for 4 min. The bacteria was then re-suspended in PBS solution and the concentration of bacteria was adjusted to OD_600_ = 1*.*0. Cements samples with Φ = 6 mm, H = 1 mm were prepared and aged in distilled water for 1 day and 7 days. The aged specimens were then placed in 96-well plates. 5 µl prepared bacteria solution was spread on the sample surface and the samples were then incubated at 37 °C for 45 min to allow the bacteria to settle on the surface. After 45 min all the samples were taken out and 135 *μ*L of TSB culture medium was added to each well. Luminescence of the media was recorded every hour by using Hidex Chameleon plate reader. Six samples were measured for each group. Growth curve of the bacteria was plotted. Luminescence of the bacteria is proportional to number of viable bacteria. That is, the material having the highest antibacterial effect will have the least viable bacteria on them.

### pH change, F^−^ and Ag^+^ release

For each group, three disc-shaped specimens (Φ = 6 mm, H = 1 mm) were molded and cured in the air for half an hour. The discs were then removed from the molds and immersed in 7.5 ml milli-Q water. The water was collected and replenished every day until day 7. The pH of the solutions was measured every day. Inducted coupled plasma-atomic emission spectroscopy (ICP-AES, Spectro Analytical Instruments, Kleve, Germany) was used to measure Ag^+^ concentration. The F^−^ concentrations were measured with a F^−^ specific electrode (6.0502.150, Metrohm, Switzerland).

### Compressive strength measurement

Six cylindrical specimens which were 4 mm in diameter and 6 mm in height were prepared for each group. The specimens were stored in 37 °C water for 1 day. The compressive strength measurement was conducted on a universal testing machine (AGS-X, Shimadzu, Japan) with a crosshead speed of 1 mm/min.

### Statistics

Statistical analysis was performed using a one-way analysis of variance (ANOVA) followed by Turkey post hoc test at *P* < 0.05 level.

## Results

### Characterization of synthesized Ag-HA and Ag-DCPA

**Fig. 1:**
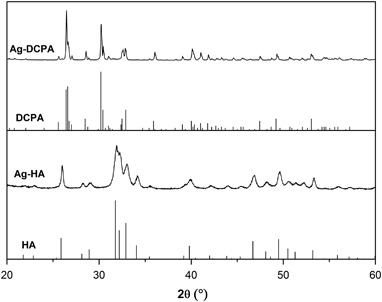
X-ray diffraction pattern of Ag-DCPA and Ag-HA prepared by precipitation method. Reference patterns shown are: PDF 01-080-6199 (HA), PDF 00-009-0080 (DCPA)

**Fig. 2:**
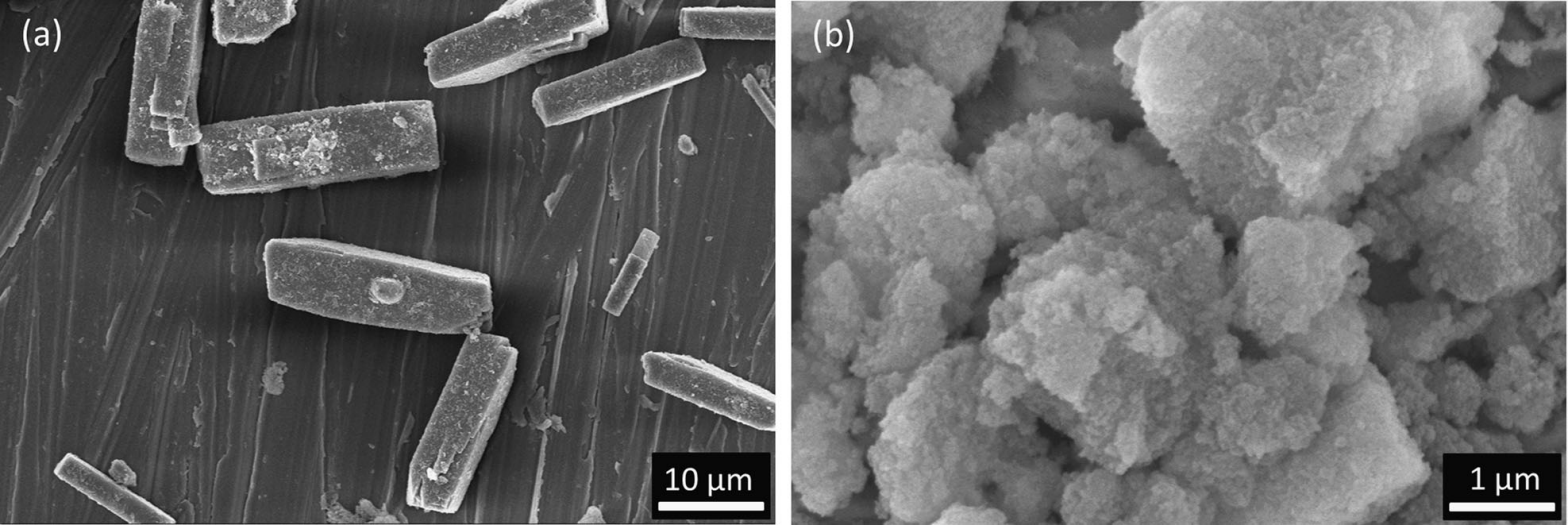
SEM images of the particles. **a** Ag-DCPA. **b** Ag-HA

XRD patterns showed Ca_10_(PO_4_)_6_(OH)_2_ was the main phase in Ag-HA and CaHPO_4_ was the main phase in Ag-DCPA, see Fig. 1. XRF analysis confirmed the existence of the Ag in the resultant particles, see Table 1. The amount of Ag in the Ag-DCPA is 0.3 wt % and 0.1 wt % in Ag-HA. The Ag-HA particle formed agglomerates while the Ag-DCPA powder was block-like, see Fig. 2. GIC modified with 10, 20 % of Ag-HA showed 0.2 and 0.3 wt % Ag respectively, while GIC modified with 10, 20 % of Ag-DCPA showed 0.4 % wt % Ag, see Table 2.

**Table 1:** Chemical composition of Ag-DCPA and Ag-HA by XRF (wt. %)

Samples	Ca	*P*	Ag	O
Ag-DCPA	33.9	22.8	0.3	43.0
Ag-HA	42.4	14.2	0.1	43.3

**Table 2:** Chemical composition of GIC modified with Ag-DCPA or Ag-HA by XRF (wt.%)

Samples	Ca	*P*	Ag	Al	Si	Sr	O
10 % Ag-DCPA	7.9	10.9	0.4	10.2	4.2	30.0	36.6
20 % Ag-DCPA	13.4	12.2	0.4	9.0	7.1	16.7	41.2
10 % Ag-HA	5.6	8.8	0.3	8.6	9.8	29.2	37.7
20 % Ag-HA	13.1	10.1	0.2	9.6	11.2	13.8	42.0

### Direct contact measurement

**Fig. 3:**
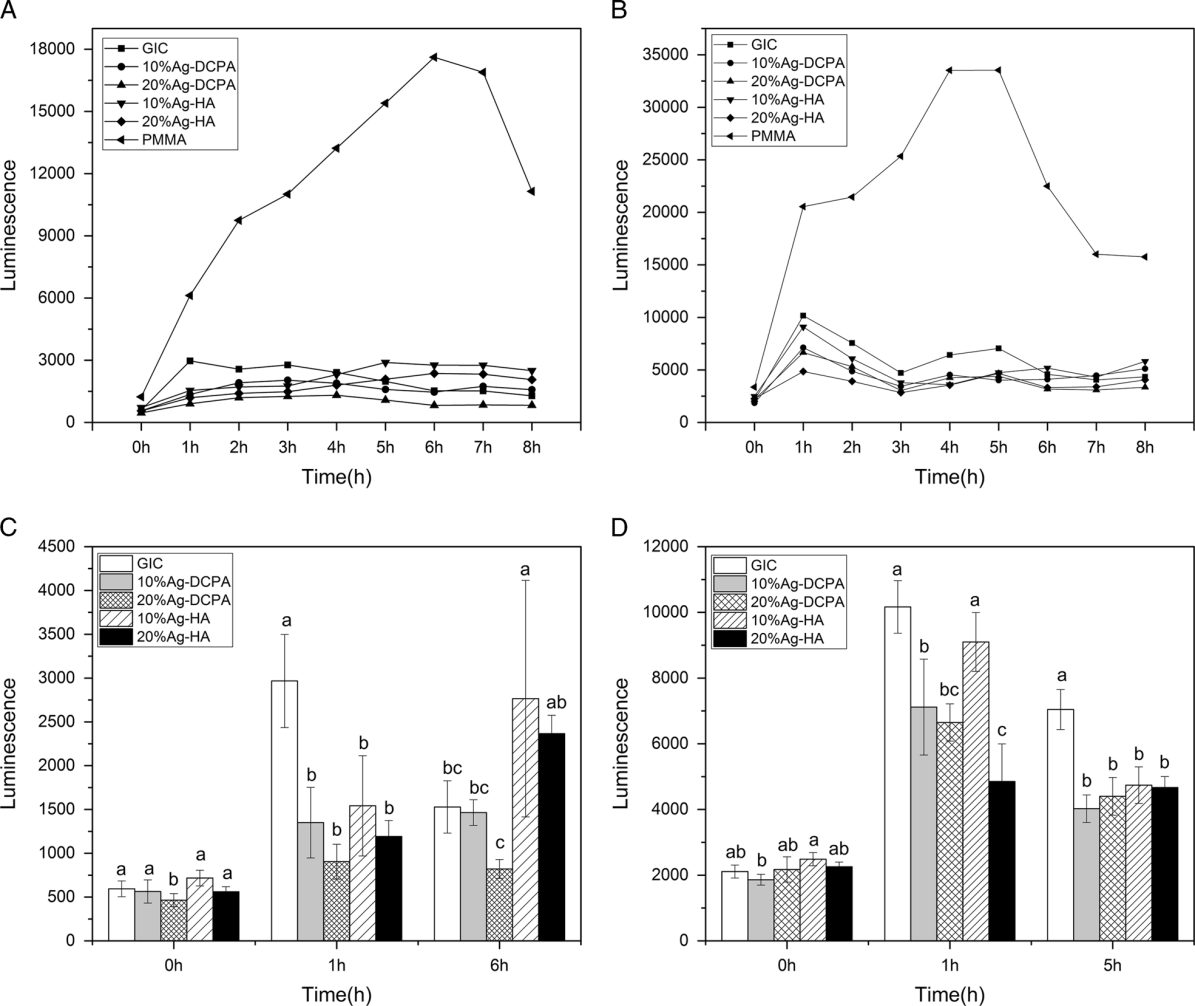
Direct contact measurement of the cements aged for 1 day and 7 days. **a** Cements aged for 1 day. **b** Cements aged for 7 days. **c** Cements aged for 1 day in selective time points after statistically analysis. **d** Cements aged for 7 days in selective time points after statistically analysis. Each point on the curve is the average of five measured wells. Groups with different letters are significantly different at *P* < 0.05 level

PMMA had the least antibacterial activity as the luminescence was seen maximum in PMMA, see Fig. 3. In case of samples aged for 1 day, 10 % Ag-HA and 20 % Ag-HA showed antibacterial effect initially but there was no sustained effect seen in them, but 20 % Ag-DCPA showed the least amount of bacteria even over a long period of time. The amount of bacteria of each group can be seen more clearly in Figs. 3c and d, in which the number of bacteria in selective time points were plot and statistically analyzed. For samples aged for 1 day, groups with Ag-HA and Ag-DCPA showed less amount of bacteria than GIC control groups (*P* < 0.05) at the first hour. However, at the sixth hour, samples with 10 % HA showed higher amount of bacteria than GIC control (*P* < 0.05). For samples aged for 7 day, the antibacterial effect of Ag-HA and Ag-DCPA was more prominent. All samples containing Ag-HA and Ag-DCPA had fewer amount of bacteria than GIC control at the first hour and the fifth hour, except samples with 10 % Ag-HA which showed equal amount of bacteria with the control at the first hour (*P* > 0.05).

### pH change, F^−^ and Ag^+^ release

**Fig. 4:**
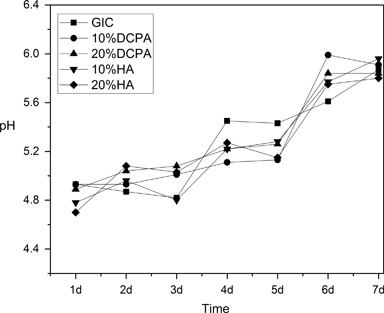
pH changes of solution over a period of 7days

**Fig. 5:**
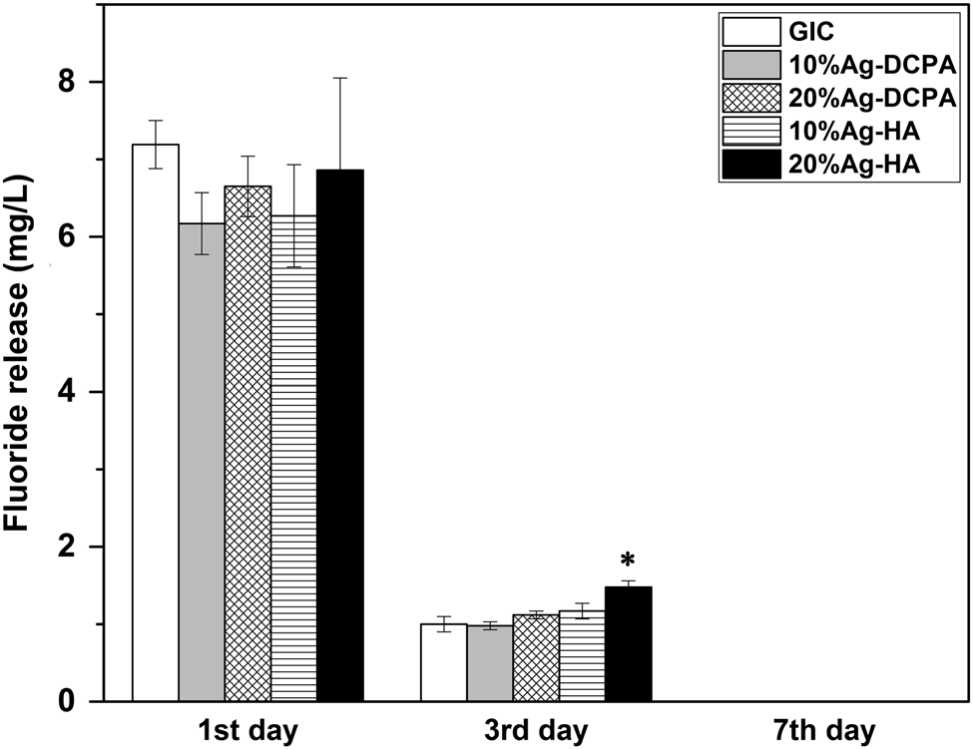
Fluoride release from different samples. * indicates significance (*P* < 0.05) between 20 % Ag-HA and all other groups

GIC showed a low pH (4.93) after one day and the pH continued to increase during the 7 days’ immersion, with the value of 5.87 on day 7, see Fig. 4. Addition of Ag-DCPA or Ag-HA didn’t change the pH. The GIC control sample showed a burst release of F^−^ on day 1 (7.2 mg/L) and the F^−^ release dramatically decreased on day 3 (1.0 mg/L), see Fig. 5. No significant difference could be observed among the different groups (*P* > 0.05), except samples with 20 % Ag-HA on day 3, which showed a little higher fluoride release (1.5 mg/L). On day 7, almost none F^−^ can be detected in all the samples. The ICP-AES results showed that concentration of Ag^+^ was under the detection limit (0.001 mg/L) for all samples.

### Compressive strength

**Fig. 6:**
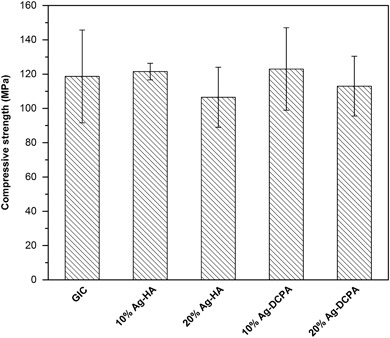
Compressive strength of the cements after storage in water for 1 day. There is no significant difference among the groups (one-way ANOVA, Tukey’s test)

There was no significant difference of compressive strength among all the groups, see Fig. 6. Compressive strength of GIC, 10 % Ag-HA, 20 % Ag-HA, 10 % Ag-DCPA and 20 % Ag-DCPA were 119 (27) MPa, 122 (5) MPa, 107 (18) MPa, 123 (24) MPa and 113 (17) MPa respectively.

## Discussion

XRF results showed that Ag exists in Ag-HA and Ag-DCPA while no Ag peak can be observed from X-ray diffraction pattern, indicating Ag^+^ could substitute some Ca^2+^ in HA and DCPA crystal structure. This is probably due to the small amount of Ag (Ca_10−*x*_Ag_*x*_(PO_4_)_6_(OH)_2_, *X* = 0.2) we doped in the experiment. In some references, the Ag peak can be observed in the X-ray diffraction pattern, showing the existence of Ag particles when the amount of doped Ag is large [16, 19]. Ag is well known as possessing antibacterial effect even since ancient times. Recent research has shown that Ag kills bacteria by increasing bacteria’s membrane permeability and production of reactive oxygen species [20]. As a promising antibacterial agent, Ag-HA was demonstrated good antibacterial properties as particles or as coatings. Kim et al. [21] synthesized HA doped with Ag, Cu and Zn and found only Ag doped HA demonstrated an obvious antimicrobial effect. Jadalannagari et al. [17] used a modified sol-gel method to synthesize Ag doped HA nano rods and antimicrobial activity was observed for all the three Ag doping concentrations. Here as shown in Fig. 3, we demonstrate Ag-HA as additives in the dental cements could improve the antibacterial of conventional GIC.

The setting of GIC is an acid-base reaction between polyelectrolyte and calciumfluoro-aluminosilicate glass [22]. Previous studies on antibacterial activities of GIC are controversial. Some studies have shown that GIC has significant antibacterial effect due to the F^−^ release or low pH [23], while others find the antibacterial effect of GIC is low [24]. In our experiment, the amount of bacteria of GIC samples is smaller than that of PMMA samples, either aged for 1 day or 7 days, as shown in Fig. 3. The incorporation of Ag-HA and Ag-DCPA can further decrease the amount of bacteria, indicating these Ag doped calcium phosphate particles can enhance the antibacterial effect of GIC. The initial pH of GIC is low, followed by a continuous increasing during the storage time, as shown in Fig. 4. The incorporation of Ag-HA and Ag-DCPA has little effect on the pH change of GIC, where we can conclude the enhanced antibacterial activity is not due to the pH change. F^−^ can reduce demineralization and enhance remineralization of enamel and dentin, which makes it widely used as anticariogenic agents in toothpaste and dental restorative materials [25]. The F^−^ release of GIC control samples significantly decreased with time, as shown in Fig. 5. Almost no F^−^ was detectable on day 7. However, incorporation of Ag-HA and Ag-DCPA had no effect on the release of F^−^. In addition, the amount of released F^−^ was low and far less than the amount that can inhibit the bacteria proliferation [26]. Brajendra et al. [27] have studied the Ag^+^ release from Ca_10−*x*_Ag_*x*_(PO_4_)_6_(OH)_2_ (0.2 ≤ *x* ≤ 0.5) and showed that *x* = 0.2 composition in phosphate buffer solution release no detectable Ag^+^ and only small amount of Ag^+^ (0.006 ppm and 0.004 ppm) can be detected in samples *x* = 0.3 and *x* = 0.5 after 72 h incubation. Even so, these Ag doped HA showed improved antibacterial property compared to un-doped HA. In our study, although no Ag^+^ can be detected in the aged solutions, GIC with Ag-DCPA and Ag-HA still showed enhanced antibacterial effect. Therefore, the enhanced antibacterial properties were unlikely due to the released Ag^+^ in the solutions. As shown in Table 2, small amount of Ag could be observed on the surfaces of modified GIC samples. Thus the enhanced antibacterial effect is probably caused by the direct contact of bacteria with Ag-DCPA and Ag-HA particles exposed on the surfaces of modified GIC samples.

Some antibacterial agents such as chlorhexidine diacetate and poly (quaternary ammonium salt) can increase the antibacterial properties of GIC but reduce the mechanical properties at the same time [28, 29]. The incorporation of Ag-HA or Ag-DCPA into the GIC did not compromise the mechanical properties of GIC, as shown in Fig. 6, indicating Ag-HA and Ag-DCPA are promising antibacterial agents for the dental cements.

## Conclusions

Ag doped monetite and hydroxyapatite particles were synthesized and characterized. The antibacterial properties of conventional glass ionomer cement could be improved by incorporation of Ag-DCPA and Ag-HA. Incorporation 10 or 20 % of these calcium phosphate particles did not compromise the compressive strength of conventional glass ionomer cement. The pH change and F^−^ ion release from glass ionomer cement in the solutions have not been influenced by the addition of Ag-DCPA and Ag-HA. The concentration of Ag^+^ was under the detection limit for all samples. The long-lasting antibacterial properties of the modified glass ionomer cement will be further investigated.
